# Basic psychological need satisfaction profiles at work: person-centered links to job crafting and intrinsic motivation

**DOI:** 10.3389/fpsyg.2026.1926358

**Published:** 2026-10-12

**Authors:** Lynelle Coxen

**Affiliations:** 1 Optentia Research Unit, North-West University, Vanderbijlpark, South Africa; 2 School of Industrial Psychology and Human Resource Management, Faculty of Economic and Management Sciences, North-West University, Vanderbijlpark, South Africa

**Keywords:** autonomy, basic psychological needs, competence, intrinsic motivation, job crafting, latent profile analysis, relatedness, self-determination theory

## Abstract

Grounded in Self-Determination Theory (SDT), this study adopted a person-centered approach to investigate whether job crafting behaviors are associated with employees’ membership in basic psychological need satisfaction profiles. Previous research has focused on contextual workplace factors such as job demands-resources and managerial behaviors, with less attention given to employees’ proactive role in influencing their motivational experiences. This study addresses this gap by identifying basic psychological need satisfaction profiles among employees in South African small and medium enterprises. It further examines whether job crafting predicts profile membership and whether these profiles differ in intrinsic motivation. A cross-sectional survey with 197 employees was conducted. They completed the Basic Psychological Need Satisfaction and Frustration Scale–Work Domain, Job Crafting Questionnaire, and Multidimensional Work Motivation Scale. Latent profile analysis supported three quantitatively differentiated profiles reflecting low, moderate, and high need satisfaction. In classification-error-adjusted analyses, job crafting dimensions were associated with profile membership. Higher task and cognitive crafting differentiated the high from the moderate profile, whereas cognitive and relational crafting differentiated the moderate from the low profile. Sensitivity analyses indicated that estimates contrasting the high and low profiles were imprecise and should be interpreted cautiously. Intrinsic motivation differed significantly across the profiles, with employees in the high need satisfaction profile reporting the highest intrinsic motivation. These findings extend person-centered research on SDT by showing that proactive job crafting behaviors are associated with employees’ membership in more adaptive need satisfaction groups.

## 1 Introduction

Self-Determination Theory (SDT) suggests that three fundamental psychological needs must be met for employees to be optimally motivated: autonomy, competence, and relatedness (Deci et al., 2017). Variable-centered research consistently shows that satisfying these needs enhances intrinsic motivation (Jungert et al., 2025; Van Den Broeck et al., 2016), which subsequently promotes employee well-being. Although variable-centered approaches have generated important insights into the effects of need satisfaction, they primarily estimate average associations within a sample (Spurk et al., 2020). As a result, they may obscure meaningful differences between employees, particularly when individuals experience varying combinations of autonomy, competence, and relatedness. To address this limitation, organizational researchers have turned to latent profile analysis (LPA), a person-centered method that identifies subgroups of employees with unique profiles (Morin et al., 2016). From a theoretical perspective, recognizing multiple profiles is important because it acknowledges that psychological needs are rarely experienced in isolation but rather in differentiated patterns.

Empirical studies confirm that basic psychological needs cluster into differentiated workplace profiles (Chen et al., 2019; Gillet et al., 2019) and that job demands and resources can predict membership in these profiles (Fernet et al., 2023; Gillet et al., 2019). However, the job demands-resources perspective emphasizes how employees are influenced by their work environment (Pirrotta et al., 2025), with less attention given to proactive behaviors that they may use to adjust their work.

Job crafting, defined as employees’ self-initiated efforts to modify their tasks, perceptions, and work relationships (Wrzesniewski and Dutton, 2001) could be seen as such proactive behaviors. By engaging in job crafting, employees may modify aspects of their work, which could subsequently be associated with outcomes such as need satisfaction in the form of better support for autonomy, competence, and relatedness. Consistent with this view, variable-centered research has demonstrated positive associations between job crafting and basic psychological need satisfaction (Slemp and Vella-Brodrick, 2013). Despite these findings, comparatively little research has examined whether job crafting predicts employees’ membership in differentiated need satisfaction profiles identified through person-centered approaches.

This represents an important gap because person-centered research on need profiles has largely focused on how external workplace conditions, such as job demands and resources and managerial behaviors, predict profile membership (Gillet et al., 2019; Huyghebaert-Zouaghi et al., 2023), studying employees as passive recipients of their work environment. Examining job crafting in relation to basic need satisfaction profiles can extend this work by examining whether employees’ proactive job crafting behaviors are associated with different profiles of autonomy, competence, and relatedness experienced at work. Building on findings that need satisfaction profiles relate to intrinsic motivation (Huyghebaert-Zouaghi et al., 2023), the present study investigates these dynamics in the underexplored context of South African small and medium enterprises (SMEs). The primary goal is to identify differentiated profiles of basic psychological need satisfaction among South African SME employees, examine the role of job crafting behaviors (Wrzesniewski and Dutton, 2001) in predicting profile membership, and explore how these profiles relate to intrinsic motivation.

In doing so, this study (a) responds to calls to advance Basic Psychological Need Theory through person-centered research (Vansteenkiste et al., 2020), (b) integrates a proactive behavioral perspective into the study of basic need profiles by considering how job crafting relates to profile membership and (c) broadens the cross-cultural application of SDT by testing these propositions in a resource-constrained South African SME context (Lynch, 2023).

## 2 Literature overview

### 2.1 Self-determination theory and basic psychological needs

Self-Determination Theory (Deci and Ryan, 2000) is a widely applied framework of human motivation that outlines the conditions under which individuals function and thrive (Deci et al., 2017). At its core, SDT argues that people have three basic psychological needs, autonomy, competence, and relatedness, that must be satisfied to foster well-being and high-quality motivation (Ryan and Deci, 2019). *Autonomy* reflects the need to act with a sense of personal choice, in which behavior is perceived as self-directed rather than driven by external pressures (Deci and Ryan, 2000). It is satisfied when individuals feel free to make choices and view their behavior as self-endorsed. *Competence* refers to the need to experience mastery and effectiveness in tasks (Deci and Ryan, 2000). It is fulfilled when individuals feel capable of performing successfully and developing skills that support growth (Van Den Broeck et al., 2016). *Relatedness* represents the need to form close, supportive, and meaningful relationships (Deci and Ryan, 2000). It is satisfied when individuals feel a sense of belonging and social value (Van Den Broeck et al., 2016). Research consistently demonstrates that satisfaction of these needs promotes well-being, performance, and motivation (Coxen et al., 2021; Ryan et al., 2022; Van Den Broeck et al., 2016). Within SDT, this is referred to as the bright motivational pathway, whereby need satisfaction facilitates optimal functioning (Vansteenkiste and Ryan, 2013). Although SDT also recognizes a complementary dark pathway, in which need frustration leads to controlled motivation and ill-being (Vansteenkiste and Ryan, 2013), the present study focuses on the bright pathway to better understand positive, growth-oriented motivational processes at work.

### 2.2 Basic psychological need profiles

Although the benefits of basic psychological need satisfaction for employee well-being are well established (Coxen et al., 2021; Van Den Broeck et al., 2016), most studies have relied on variable-centered approaches. More recently, person-centered techniques such as LPA have been used to uncover heterogeneity in need experiences. Much of this work has been conducted in educational settings (Bérubé-Deschênes et al., 2026; Li et al., 2021), whereas organizational applications remain limited. Fernet et al. (2023), for example, identified five employee profiles spanning work and personal life, ranging from globally satisfied to globally unsatisfied, as well as misaligned patterns across domains (e.g., high autonomy, low competence). Similarly, organizational studies by Gillet et al. (2019) and Huyghebaert-Zouaghi et al. (2023) found profiles characterized by both high and low global need satisfaction, as well as mixed configurations in which some needs were satisfied while others were frustrated or unfulfilled. Although a normative profile of moderate need satisfaction is sometimes identified, the majority of employees tend to cluster into profiles characterized by either relatively high or relatively low need satisfaction, suggesting that need experiences at work are more polarized than uniform (Fernet et al., 2023; Gillet et al., 2019; Huyghebaert-Zouaghi et al., 2022). These configurations predict differentiated motivational pathways, underscoring the importance of tailoring managerial support to employees’ need states. Based on prior person-centered research on basic psychological needs, employees are expected to form profiles that differ in their levels of need satisfaction. The following research question is therefore posed:

*Research Question (RQ)*: What profiles of basic psychological need satisfaction emerge among South African SME employees?

### 2.3 Job crafting as a statistical predictor of need profile membership

Job crafting refers to the proactive behaviors through which employees modify the physical, cognitive, and social dimensions of their work to better align with their skills, interests, and needs (Slemp and Vella-Brodrick, 2013). *Cognitive crafting* involves reinterpreting the meaning of work to enhance its personal significance, for example, by perceiving one’s role as contributing to a broader purpose (Slemp and Vella-Brodrick, 2013). *Relational crafting* entails altering or developing workplace interactions, for example, by fostering stronger relationships with colleagues who share similar interests (Slemp and Vella-Brodrick, 2013). *Task crafting* involves intentionally changing the number, scope, or type of work activities performed, such as by developing more efficient processes (Slemp and Vella-Brodrick, 2013). These behaviors are self-initiated rather than management-directed, reflecting employees’ active role in influencing their work environment (Slemp and Vella-Brodrick, 2013).

A recent scoping review indicates that each form of job crafting is positively associated with the fulfillment of the basic psychological needs (Napier et al., 2024). *Task crafting* enables employees to modify aspects of their work to better align with their needs, values, and skill sets, which may be associated with greater feelings of autonomy and competence (Petrou et al., 2015; Slemp et al., 2015; Tims et al., 2012). *Relational crafting* has been associated with greater relatedness satisfaction by enabling employees to develop and strengthen workplace relationships (Napier et al., 2024) and may also relate to autonomy by providing choice in social engagement. *Cognitive crafting* may support autonomy by helping employees internalize work goals, and may also strengthen competence and relatedness by reinforcing a sense of efficacy and connection in relation to others (Olafsen et al., 2025).

Although previous research has consistently demonstrated positive associations between job crafting and basic psychological need satisfaction (Slemp and Vella-Brodrick, 2014; Toyama et al., 2022), most studies have adopted a variable-centered approach, focusing on average relationships between constructs. In contrast, a person-centered approach recognizes patterns of how employees satisfy their needs for autonomy, competence, and relatedness, resulting in need satisfaction profiles. A recent study has applied such a person-centered approach, showing that job crafting in the form of increasing job resources and decreasing job demands predicts membership in basic psychological need satisfaction profiles (Huyghebaert-Zouaghi et al., 2022). However, their study was based on a job demands–resources perspective of job crafting and focused on a single occupational group. As a result, it remains unclear whether other forms of crafting identified in the literature (i.e., task, relational, and cognitive crafting) are similarly associated with differentiated profiles of basic psychological needs across a broader range of work settings. To address this gap, the present study draws on the job crafting framework proposed by Wrzesniewski and Dutton (2001) and examines employees working in SMEs across multiple sectors. In doing so, it investigates whether task, relational, and cognitive job crafting relate to membership in basic psychological need profiles in a broader occupational and organizational context. In SMEs, where organizational systems and human resource management practices tend to be less formalized (Storey et al., 2010), employees may rely more heavily on self-initiated strategies such as job crafting to create need-supportive work conditions; therefore, examining SMEs provides a theoretically meaningful test of whether crafting predicts need satisfaction profile membership beyond the healthcare context.

Given that job crafting reflects self-initiated efforts to align work characteristics with personal motives and preferences, employees who engage more frequently in crafting behaviors are expected to report greater satisfaction across all three needs, increasing the likelihood that they will belong to profiles characterized by higher need satisfaction. Conversely, employees who engage less in crafting behaviors are expected to be more likely to belong to profiles characterized by lower need satisfaction. The following hypothesis is therefore proposed:

*Hypothesis 1 (H1):* SME employees who report higher levels of (a) task, (b) relational, and (c) cognitive job crafting will be more likely to be classified into profiles with higher basic psychological need satisfaction.

### 2.4 Intrinsic motivation as a distal outcome of need profiles

Motivation, often defined as the force that initiates, directs, and sustains behavior, is a central driver of employee well-being and performance (Pinder, 2008). SDT distinguishes between different types of motivation. On one end of the continuum are controlled forms, influenced by external rewards, social pressures, or guilt; on the other are more autonomous forms, including intrinsic motivation (Ryan and Deci, 2019). A recent meta-analysis highlighted that *intrinsic motivation*, characterized by engaging in tasks out of genuine interest and enjoyment, has particularly positive implications for employees, being strongly associated with well-being outcomes (Van Den Broeck et al., 2021). Intrinsically motivated employees tend to show higher levels of engagement, job satisfaction, and proactivity, while being less likely to experience burnout, turnover intentions, and absenteeism (Van Den Broeck et al., 2021).

SDT studies on employees have consistently shown that the satisfaction of basic psychological needs leads to higher levels of intrinsic motivation (see Van den Broeck et al., 2016, 2021, for a review). This is because when employees have control over their work (*autonomy*), feel competent in reaching work outcomes (*competence*), and feel connected to others they work with (*relatedness*), they are more likely to engage in their work out of genuine interest rather than external obligation (Van Den Broeck et al., 2021). Using a necessity-logic approach, Ding and Kuvaas (2025) demonstrated that intrinsic motivation is especially constrained by need satisfaction. That is, need satisfaction functions as a necessary condition for intrinsic motivation to occur.

Intrinsic motivation was therefore selected as the distal outcome in this study due to its central role in SDT as the most self-determined form of motivation and its well-established dependence on need satisfaction. Since latent profiles represent different combinations of autonomy, competence, and relatedness satisfaction within employees, they should yield different levels of intrinsic motivation. Profiles characterized by high need satisfaction provide the most favorable conditions for intrinsic motivation to emerge, while profiles characterized by low need satisfaction constrain it. This reasoning is consistent with person-centered SDT research showing that employees’ levels of motivational self-determination differ significantly across need-state profiles (Huyghebaert-Zouaghi et al., 2023). Building on this theoretical foundation, the following hypothesis is proposed:

*Hypothesis 2 (H2):* SME employees in profiles characterized by higher basic psychological need satisfaction will report higher levels of intrinsic motivation than those in profiles characterized by lower need satisfaction.

A related manuscript based on the same dataset examines the indirect effects of basic psychological needs in the relationship between job crafting and intrinsic motivation using a variable-centered approach (De Kock et al., 2026). In contrast, the present study uses a person-centered approach to identify empirically differentiated need satisfaction profiles and examines whether job crafting dimensions predict profile membership. Thus, although the studies use the same dataset, they address different research questions using different analytical approaches and outcomes.

## 3 Materials and methods

### 3.1 Research approach and design

A quantitative, cross-sectional survey design was adopted, well-suited to the study’s aim of uncovering latent profiles of basic psychological need satisfaction among South African SME employees at a single point in time. Widely recognized in social science research, the cross-sectional design enables researchers to provide a snapshot of variables and their relationships at a specific point in time (Wang and Cheng, 2020). Although this design does not permit causal inference or the examination of changes over time, it remains valuable for identifying important patterns and associations among variables and for generating hypotheses to inform future research and interventions (Wang and Cheng, 2020).

### 3.2 Participants

The final sample consisted of (*n* = 197) South African employees working in SMEs. SMEs, defined as businesses employing between 11 and 250 employees (De Wet, 2019) play a significant role in the economy and constitute the majority of private businesses in South Africa (Kalidas et al., 2020). They were sampled using non-probability purposive and snowball sampling. Of the participants included in the study, 66.5% were female (*n* = 131) and 33.5% male (*n* = 66). Participants’ age ranged from 18 to 73 years (*M* = 38.12, SD = 13.33). Educational levels varied, with 31% having secondary education, 30% holding higher education qualifications and 20.3% possessing postgraduate degrees. A small proportion (4.6%) did not disclose their education level. In terms of ethnicity, 67.5% identified as White, 22.3% as African, 4.6% as Colored, 4.6% as Indian, and 0.5% as Asian, while 0.5% preferred not to disclose. Tenure ranged from 4 months to 52 years (M = 158.77 months, SD = 155.96). Most participants were employed on-site (73.6%), with 18.3% working in hybrid arrangements and 8.1% working online. Employment spanned various sectors, with the largest group in finance, real estate, and business services (34.0%). Other sectors included community, social and personal services (14.2%), retail, motor trade and commercial (12.7%), manufacturing (10.2%), and transport, storage, and communication (8.1%). Smaller proportions were found in catering, accommodation, and other trade (4.6%), wholesale (4.1%), mining and quarrying (3.6%), construction (3.0%), agents and allied services (3.0%), and agriculture, forestry, and fishing (1.5%). Only 1.0% of participants did not indicate their sector.

### 3.3 Measuring instruments

A biographical questionnaire was used to collect demographic data from participants, including gender, age, educational level, ethnicity, tenure, working arrangements, and sector.

#### 3.3.1 Basic psychological needs

Basic psychological needs were assessed using the Basic Psychological Need Satisfaction and Frustration Scale – Work Domain (BPNSFS; Chen et al., 2015; Schultz et al., 2015). The need satisfaction subscale consists of 12 items, measuring autonomy, competence, and relatedness satisfaction with four items each. Responses were recorded on a seven-point Likert-type scale ranging from 1 (*strongly disagree*) to 7 (*strongly agree*). Example items include: “At work, I feel that I do what really interests me” (*autonomy satisfaction*); “At work, I feel capable at what I do” (*competence satisfaction*); and “At work, I feel that the people I care about also care about me” (*relatedness satisfaction*). The basic psychological need satisfaction subscales have demonstrated acceptable reliability with coefficients ranging from 0.79 to 0.87 across 32 countries, including South Africa (Grubbs et al., 2025).

#### 3.3.2 Job crafting

Job crafting was measured using the Job Crafting Questionnaire (JCQ; Slemp and Vella-Brodrick, 2013). The questionnaire consists of 15 items, measuring cognitive, task, and relational crafting with five items each. Responses were recorded on a six-point Frequency scale ranging from 1 (*hardly ever*) to 6 (*very often*). For example, employees were asked how often they: “Change the scope or types of tasks that you complete at work” (*task crafting*), “Reflect on the role your job has for your overall well-being” (*cognitive crafting*), and “Choose to mentor new employees (officially or unofficially)” (*relational crafting*). The job crafting subscales have demonstrated acceptable reliability in a previous South African study, with coefficients ranging from 0.71 to 0.85 (Munnisunker and Dhanpat, 2025).

#### 3.3.3 Intrinsic motivation

Intrinsic motivation was measured using the Multidimensional Work Motivation Scale (MWMS; Gagné et al., 2015). The intrinsic motivation subscale consists of 3 items and uses a seven-point scale ranging from 1 (*not at all*) to 7 (*completely*). The stem is “Why do you or would you put effort into your current job?” An example item is: “Because I have fun doing my job”. The intrinsic motivation subscale have shown acceptable reliability in a previous South African study (Coxen et al., 2023).

### 3.4 Research procedure

Ethical approval for the study was obtained from the institutional research ethics committee (NWU-01724-24-A4). Data were collected using an online survey administered through Google Forms. Recruitment was conducted via an advertisement featuring a QR code that linked participants to the information sheet, informed consent form, and surveys. The advertisement was distributed via social media platforms, personal networks, and SMEs and business chambers across South Africa.

The information sheet provided details regarding the purpose of the study, participation requirements, potential benefits, and assurances of anonymity and voluntary participation. It also included the researchers’ contact details for any questions or feedback requests. Participants were required to provide electronic informed consent before proceeding to the survey. They then completed demographic questions, followed by the study measures, and no identifying information was collected. The measures were administered in English, the language most commonly used in SME contexts. Additionally, the minimum requirement that participants have a Grade 12 qualification ensures they are sufficiently proficient in English. The online format allowed participants to complete the survey at a time and place convenient to them. Participants were informed that they could withdraw from the study at any stage without penalty and all data were securely stored in accordance with the provisions of the Protection of Personal Information Act (POPIA) of South Africa. Data were collected between August 2024 and November 2025.

A total of 197 submitted questionnaires were retained for analysis. Because Google Forms recorded only submitted questionnaires, the number of surveys opened but not completed was unavailable. All survey items were mandatory, therefore, no item-level missing data were present. Duplicate submissions were checked, and none were identified.

### 3.5 Data analysis

SPSS Version 30 (IBM Corp., 2024) and Mplus 8.11 (Muthén and Muthén, 1998) were used for data analysis. Descriptive statistics were used to summarize participants’ characteristics and examine the distributions of the study variables. Reliability was calculated using the Omega coefficient (ω), with a cut-off value of >0.70 (Wang and Wang, 2020). Pearson’s correlations were used to determine the relationships between the variables. Cohen’s (2013) effect sizes were used as indicators of practical significance: small effect (>0.10), medium effect (>0.30), and large effect (>0.50).

Confirmatory factor analysis (CFA) was conducted to determine the factor structure of job crafting, basic psychological need satisfaction, and intrinsic motivation. In line with Kline’s (2016) guidelines, model fit was assessed with the following indices: comparative fit index (CFI), Tucker–Lewis index (TLI), root mean square error of approximation (RMSEA), and standardized root mean square residual (SRMR). Recommended thresholds were values above 0.90 for CFI and TLI and <0.08 for RMSEA and SRMR (Kline, 2016).

Latent profile analysis (LPA), using the maximum likelihood with robust standard errors (MLR) estimator, was conducted using factor scores as indicators. Model retention was determined by whether adding profiles significantly improved the fit compared to the reference model. Competing models were evaluated using the Bayesian information criterion (BIC), the Akaike information criterion (AIC), and the sample-size-adjusted BIC (ABIC) (Kline, 2016; Wang and Wang, 2020). The optimal number of profiles was further determined using the Lo–Mendell–Rubin likelihood ratio test (LMR-LRT), the adjusted LMR test (aLMR), and the bootstrapped likelihood ratio test (BLRT) (Lo, 2001; Wang and Wang, 2020). Entropy values (ranging from 0 to 1) were examined to assess classification accuracy, with values closer to 1 reflecting stronger separation between profiles (Pastor et al., 2007). In addition, average latent profile probabilities were considered, with probabilities above 0.80 indicating adequate classification quality (Spurk et al., 2020). Since there are no universally accepted sample size requirements for LPA, profile adequacy was evaluated using these classification and model fit indices in conjunction with theoretical interpretability, rather than sample size alone.

Whether the different types of job crafting predict latent profile membership was evaluated using the R3STEP approach, which incorporates multinomial logistic regression and adjusts for classification uncertainty (Vermunt, 2010). Differences in intrinsic motivation across the identified profiles were examined with the BCH procedure, a method that enables unbiased comparisons of continuous distal outcomes while maintaining profile separation (Asparouhov and Muthén, 2014).

## 4 Results

### 4.1 Confirmatory factor analysis

A measurement model comprising five indicators per job crafting dimension (task, cognitive, and relational crafting), four indicators per basic psychological need (autonomy, competence, and relatedness), and three indicators for intrinsic motivation was tested using CFA. The measurement model demonstrated adequate overall fit: *χ*^2^ = 895.45 (*df* = 384), *p* < 0.001; CFI = 0.95; TLI = 0.94; RMSEA = 0.08 (90% CI [0.08, 0.09]); and SRMR = 0.06. Findings supported the hypothesized three-factor structure for job crafting, three-factor structure for basic psychological needs, and one-factor structure for intrinsic motivation. Standardized factor loadings ranged from 0.73 to 0.81 for autonomy, 0.87 to 0.93 for competence, 0.78 to 0.94 for relatedness, 0.48 to 0.89 for task crafting, 0.70 to 0.89 for cognitive crafting, 0.59 to 0.84 for relational crafting, and 0.90 to 0.96 for intrinsic motivation. Although one task crafting item showed a relatively low loading (*λ* = 0.48, *p* < 0.001), its removal did not improve model fit; it was therefore retained to preserve the theoretical integrity and content validity of the construct.

To examine whether the three basic psychological needs could be represented by a single general need satisfaction construct, an alternative one-factor CFA was estimated. The one-factor model demonstrated poor fit: *χ*^2^ = 312.37 (*df* = 54), *p* < 0.001; CFI = 0.66; TLI = 0.59; RMSEA = 0.16 (90% CI [0.14, 0.17]), whereas the hypothesized three-factor model provided substantially better fit. These findings support the empirical distinction between autonomy, competence, and relatedness.

### 4.2 Descriptive statistics, reliabilities and correlations

The descriptive statistics (means and standard deviations), McDonald’s Omega reliability coefficients, and correlations are reported in Table 1. All scales demonstrated acceptable reliability, ranging from 0.77 to 0.94, exceeding the 0.70 cut-off. The correlations among all variables were statistically significant, with medium to large effect sizes. The three basic psychological needs were positively related to each other and to intrinsic motivation, with large effects, consistent with SDT research. They were also positively related to the three types of job crafting, with large effects, except for the relationship between relatedness and task crafting, which showed a medium effect. Finally, the three job crafting dimensions were positively associated with intrinsic motivation, with large effects.

**Table 1:** Descriptive statistics, reliabilities, and correlations of scales.

Variable	*ω*	*M*	SD	1	2	3	4	5	6
1. Autonomy	0.77	3.69	0.80	–	–	–	–	–	–
2. Competence	0.89	4.10	0.71	0.83^‡**^	–	–	–	–	–
3. Relatedness	0.89	3.75	0.77	0.80^‡**^	0.59^‡**^	–	–	–	–
4. Task crafting	0.82	4.30	0.98	0.64^‡**^	0.65^‡**^	0.35^†**^	–	–	–
5. Cognitive crafting	0.85	4.49	1.04	0.69^‡**^	0.72^‡**^	0.55^‡**^	0.67^‡**^	–	–
6. Relational crafting	0.82	4.09	1.13	0.72^‡**^	0.60^‡**^	0.76^‡**^	0.62^‡**^	0.73^‡**^	–
7. Intrinsic motivation	0.94	4.97	1.28	0.85^‡**^	0.69^‡**^	0.47^†**^	0.61^‡**^	0.63^‡**^	0.50^‡**^

ω = McDonald’s omega coefficient; M = Mean; SD = Standard Deviation; ^**^p < 0.01. Factor scores saved from the measurement model were used to compute the correlations. For practical significance: ^†^*r* ≥ 0.30; ^‡^*r* ≥ 0.50.

### 4.3 Latent profile analysis

The LPA was conducted on the three basic psychological needs to determine whether the sample had different need-experience profiles using factor scores from the measurement model. Since the exact number of latent profiles is unknown and cannot be directly determined from the model, several models with varying numbers of latent profiles were evaluated. As recommended by Wang and Wang (2020), the process began with a single-profile model, and the number of profiles was increased incrementally by one for each subsequent model. The results of five competing models are displayed in Table 2.

**Table 2:** Comparisons of different basic psychological needs latent profile analysis models.

Profiles	AIC	BIC	aBIC	Entropy	LMR LR test *p*-value	aLMR LR test *p*-value	BLRT *p*-value	Smallest profile membership
1	1343.33	1363.03	1344.02	–	–	–	–	–
2	1164.96	1197.79	1166.11	0.80	0.001	0.002	<0.001	33.5%
3	1031.48	1077.44	1033.09	0.90	<0.001	<0.001	<0.001	14.7%
4	1000.71	1059.81	1002.79	0.92	0.31	0.32	<0.001	2.03%
5	986.70	1058.93	989.23	0.88	0.03	0.03	0.18	0.5%

AIC = Akaike information criterion; BIC = Bayesian information criterion; aBIC = adjusted Bayesian information criterion; LMR LR = Lo–Mendell–Rubin test; aLMR LR = adjusted Lo–Mendell–Rubin test; BLRT = bootstrapped likelihood ration test.

Model fit indices suggest that the three-profile model provides the best fit and the most parsimony. Compared to the two-profile model, the three-profile model shows a substantial decrease in AIC, BIC, and aBIC values (ΔAIC = 133.48; ΔBIC = 120.35; and ΔaBIC = 133.02). In addition, the entropy value of 0.90 suggests good classification accuracy and is the highest across all models. Finally, the LMR, aLMR, and BLRT tests are all statistically significant (*p* < 0.001), supporting the three-profile model over models with fewer classes.

Although the four- and five-profile models showed lower AIC, BIC, and aBIC values than the three-profile model, only a small number of people belonged to the models’ smallest profile (2.03 and 0.5%, respectively). The smallest profile in the three-profile solution accounted for 14.7% of the sample, indicating adequate class separation. This proportion exceeds the 10% benchmark referenced in Sinha et al. (2021), often used as a general guideline for evaluating profile stability. This model also replicated the best log-likelihood value (−501.74) several times using the default number of starting values. A large number of random starts (2000 initial and 500 final optimizations) were used to ensure model convergence and replicate the best log-likelihood value.

### 4.4 Local independence assessment

Local independence was evaluated by inspecting within-class correlations among the latent profile indicators (autonomy, competence and relatedness). Several within-class correlations exceeded the recommended 0.30 threshold (Visser and Depaoli, 2022) particularly between autonomy and competence, and between autonomy and relatedness, suggesting residual associations not fully explained by the latent classes. To account for this, the three-class LPA model was re-estimated with residual covariances freely estimated between autonomy and competence as well as autonomy and relatedness (Asparouhov and Muthén, 2014). Both residual covariances were statistically significant (Autonomy–Competence: *β* = 0.056, *p* < 0.001; Autonomy–Relatedness: *β* = 0.153, *p* < 0.001), indicating meaningful local dependence. Allowing these residual covariances substantially improved model fit relative to the local independence model (e.g., AIC = 911.76 vs. 1031.48; BIC = 964.29 vs. 1077.44; Log likelihood = −439.88 vs. −501.74). The residual covariance between competence and relatedness was not statistically significant (*p* = 0.96) and was therefore not retained. The class structure remained substantively equivalent, with profiles representing low, moderate, and high levels of need satisfaction.

To confirm the selected solution after accounting for local dependence, the two-, three-, four-, and five-profile models were re-estimated using the revised specification. The three-profile solution remained preferred based on profile size, classification quality, parsimony, and substantive interpretability. Although the four- and five-profile models had lower information criteria, they produced small profiles (6.5 and 3.0%, respectively). The three-profile solution therefore provided the most interpretable and parsimonious solution. It reflected different levels of need satisfaction in terms of autonomy, competence, and relatedness. Profile 1 represented individuals with low need satisfaction (17.8%; *n* = 35), whereas Profile 2 reflected high levels (11.7%; *n* = 23). Profile 3 showed moderate need satisfaction (70.6%; *n* = 139) across indicators. The profiles thus differed primarily in the degree, rather than the pattern, of need satisfaction, suggesting that the three needs tend to covary. Figure 1 shows the three profiles. The average latent class assignment probabilities for individuals assigned to each profile were 0.96 (Profile 1), 0.93 (Profile 2) and 0.94 (Profile 3). These values indicate that participants were classified into their most likely latent profile with high certainty. Classification accuracy remained high (entropy = 0.87).

**Figure 1:**
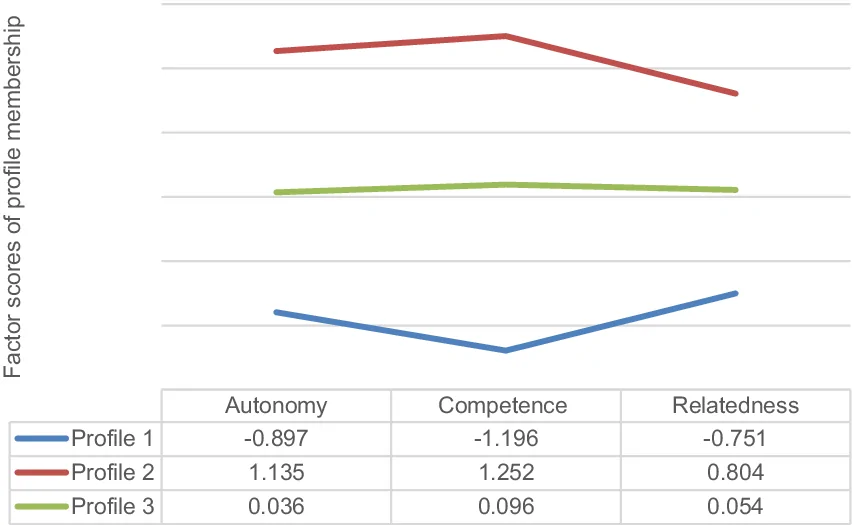
Three profiles of basic psychological need satisfaction.

### 4.5 Job crafting as predictors of profile membership

In the final three-profile solution, task, cognitive, and relational crafting were entered simultaneously as predictors of profile membership using the R3STEP procedure. Prior to estimating the multinomial logistic regression, multicollinearity diagnostics were examined. Tolerance values ranged from 0.66 to 0.72 and variance inflation factors ranged from 1.38 to 1.53, indicating no evidence of problematic multicollinearity. Table 3 presents the findings from this multinomial logistic regression analysis. Results showed that higher levels of task crafting were associated with a greater likelihood of belonging to the high need satisfaction profile than the low need satisfaction profile (OR = 71.72, *p* < 0.01). Cognitive crafting was also associated with a greater likelihood of belonging to the high rather than the low need satisfaction profile (OR = 335.42, *p* < 0.001) and the moderate rather than the low need satisfaction profile (OR = 12.70, *p* < 0.05). In addition, higher levels of relational crafting were associated with a greater likelihood of belonging to the high rather than the low need satisfaction profile (OR = 5.44, *p* < 0.05) and the moderate rather than the low need satisfaction profile (OR = 3.40, *p* < 0.05).

**Table 3:** The three-step procedure results for antecedents (R3STEP).

Predictors	High vs. Low			Moderate vs. Low			High vs. Moderate		
	Est. (S. E.)	OR	[95% CI]	Est. (S. E.)	OR	[95% CI]	Est. (S. E.)	OR	[95% CI]
Task crafting	4.27 (1.47)^**^	71.72	[3.99; 1,290.26]	1.85 (1.27)	6.36	[0.53; 76.62]	2.42 (0.76)^**^	11.29	[2.55; 49.98]
Cognitive crafting	5.82 (1.67)^***^	335.42	[12.70; 8,857.38]	2.54 (1.27)^*^	12.70	[1.06; 152.27]	3.27 (1.08)^**^	26.40	[3.16; 220.72]
Relational crafting	1.70 (0.74)^*^	5.44	[1.28; 23.21]	1.23 (0.53)^*^	3.40	[1.21; 9.57]	0.47 (0.53)	1.60	[0.57; 4.50]

Positive coefficients indicate that higher values on the predictor are associated with a greater likelihood of membership in the first profile named in each comparison, relative to the second profile. Est. = estimate (β) from the R3STEP multinomial logistic regression; S. E. = standard error; OR = Odds Ratio; 95% CI = 95% Confidence Intervals; ^*^p < 0.05, ^**^p < 0.01, ^***^p < 0.001.

When comparing the high and moderate need satisfaction profiles, higher levels of task crafting were associated with a greater likelihood of belonging to the high rather than the moderate profile (OR = 11.29, *p* < 0.01). Similarly, higher levels of cognitive crafting were associated with a greater likelihood of belonging to the high rather than the moderate profile (OR = 26.40, *p* < 0.01). Relational crafting did not significantly distinguish between membership in the high and moderate need satisfaction profiles (OR = 1.60, *p* = 0.37). Overall, higher levels of task, cognitive, and relational crafting were associated with a greater likelihood of membership in the high relative to the low need satisfaction profile. Cognitive and relational crafting were additionally associated with a greater likelihood of membership in the moderate relative to the low profile. When distinguishing between the high and moderate profiles, task and cognitive crafting were significant predictors, whereas relational crafting was not. These findings provide support for Hypothesis 1.

Given the relatively large odds ratios observed in the R3STEP analysis, a *post hoc* sensitivity analysis using Firth penalized logistic regression was conducted to assess the potential influence of sparse data and quasi-complete separation. Separation can result in unstable estimates in logistic and multinomial logistic regression, particularly when some outcome groups are small or predictors strongly distinguish between groups (Cook et al., 2018). The analyses estimated pairwise binary comparisons of the low and high need satisfaction profiles with the moderate need satisfaction profile, using most-likely profile membership as the outcome and task, cognitive, and relational crafting as predictors. Firth’s penalized-likelihood approach reduces small-sample bias and yields finite estimates in the presence of complete or quasi-complete separation (Heinze and Schemper, 2002). These analyses were interpreted as robustness checks because they did not incorporate the classification-error adjustment used in the primary R3STEP analysis. Table 4 presents the results of the analysis.

**Table 4:** Firth penalized logistic-regression sensitivity analyses of job crafting as predictors of most-likely need-satisfaction profile membership.

Predictors	High vs. Low			Moderate vs. Low			High vs. Moderate		
	Est. (S. E.)	OR	[95% CI]	Est. (S. E.)	OR	[95% CI]	Est. (S. E.)	OR	[95% CI]
Task crafting	1.66 (1.47)	5.25	[0.03; 251.38]	1.19 (0.50)^*^	3.29	[1.23; 9.51]	1.96 (0.75)^**^	7.12	[1.62; 37.88]
Cognitive crafting	1.79 (1.52)	5.99	[0.25; 269,537.95]	1.47 (0.58)^**^	4.34	[1.42; 14.88]	2.51 (0.78)^***^	12.30	[2.79; 69.04]
Relational crafting	4.20 (1.98)	66.80	[0.14; 199,142.31]	1.01 (0.45)^*^	2.74	[1.09; 7.01]	0.22 (0.51)	1.25	[0.46; 3.89]

Firth penalized logistic-regression sensitivity analyses predicting most-likely need-satisfaction profile membership from job crafting. Low need satisfaction = Class 1 (𝑛=35); High need satisfaction = Class 2 (𝑛=23); Moderate need satisfaction = Class 3 (𝑛=139). Each model included task crafting, cognitive crafting, and relational crafting simultaneously. Est. = estimated penalized log-odds coefficient; S. E. = standard error; OR = Odds Ratio; 95% CI = 95% Confidence Intervals; ^*^p < 0.05, ^**^p < 0.01, ^***^p < 0.001.

The first named profile was coded as the event category in each comparison. Because these models used most-likely profile membership, they did not account for classification uncertainty and were interpreted as sensitivity analyses alongside the primary R3STEP findings.

In the moderate versus low need satisfaction comparison, task crafting (OR = 3.29, 95% CI [1.23, 9.51], 𝑝 < 0.05), cognitive crafting (OR = 4.34, 95% CI [1.42, 14.88], 𝑝 < 0.01) and relational crafting (OR = 2.74, 95% CI [1.09, 7.01], 𝑝 < 0.05) were significant predictors. In the high versus moderate need satisfaction comparison, task crafting (OR = 7.12, 95% CI [1.62, 37.88], 𝑝 < 0.01) and cognitive crafting (OR = 12.30, 95% CI [2.79, 69.04], 𝑝 < 0.001) were significant, whereas relational crafting was not (OR = 1.25, 95% CI [0.46, 3.89], 𝑝 = 0.67). In contrast, none of the predictors significantly distinguished the high from the low need satisfaction profile, and the wide confidence intervals indicated considerable imprecision, consistent with sparse-data instability and possible quasi-complete separation. Thus, the sensitivity analyses generally supported the stability of the findings for both the moderate versus low and high versus moderate comparisons. However, the high versus low comparison should be interpreted with caution.

### 4.6 Intrinsic motivation as a distal outcome of profile membership

Finally, a BCH analysis was conducted with intrinsic motivation as a distal outcome of latent profile membership (see Figure 2). Intrinsic motivation was represented by saved standardized factor scores derived from the CFA. The overall test indicated significant mean differences among the profiles [*χ*^2^(2) = 214.09, *p* < 0.001]. Intrinsic motivation was highest in the high need satisfaction profile (*M* = 1.30; SE = 0.12), moderate in the moderate profile (*M* = 0.02; SE = 0.06), and lowest in the low profile (*M* = −0.97; SE = 0.10). Pairwise BCH comparisons indicated significant differences between the low and high profiles [*χ*^2^(1) = 212.29, *p* < 0.001] with a standardized mean difference of 2.27 SD, the low and moderate profiles [*χ*^2^(1) = 69.57, *p* < 0.001] with a standardized mean difference of 0.99 SD and the high and moderate profiles [*χ*^2^(1) = 81.56, *p* < 0.001] with a standardized mean difference of 1.28 SD. These findings are consistent with SDT, suggesting that employees who experience greater satisfaction of their basic psychological needs report higher intrinsic motivation. Accordingly, Hypothesis 2 was supported.

**Figure 2:**
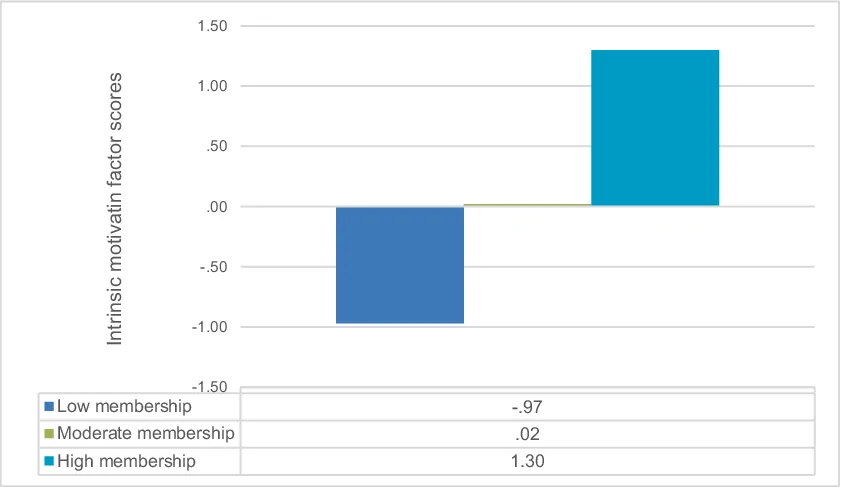
Standardized intrinsic motivation factor scores across basic psychological need satisfaction profiles. Values represent BCH-estimated means of standardized CFA-derived intrinsic-motivation factor scores (*M* = 0, SD = 1in the full sample). Higher values indicate higher intrinsic motivation. The low, moderate, and high need satisfaction profiles differed significantly from one another (all *p* < 0.001).

## 5 Discussion

Responding to calls to advance SDT through person-centered approaches (Vansteenkiste et al., 2020), this study identified subgroups of basic psychological need satisfaction experiences among South African SME employees. The study further examined job crafting as a predictor of profile membership and investigated whether the profiles differed in terms of intrinsic motivation. While SDT research has extensively focused on social-contextual antecedents of need satisfaction, less attention has been devoted to individual-level behavioral factors that may also determine employees’ motivational experiences (Ryan and Deci, 2019, 2020, 2022).

The LPA revealed three profiles that differed quantitatively in employees’ overall level of need satisfaction, demonstrating differences in the extent to which autonomy, competence, and relatedness were jointly satisfied. Consistent with prior research (Fernet et al., 2023; Gillet et al., 2019; Huyghebaert-Zouaghi et al., 2023), the present study found globally high, moderate, and low need satisfaction profiles. However, unlike these previous studies, no mixed configurations emerged (e.g., high autonomy but low competence). Instead, the three basic psychological needs shifted together, forming profiles that differed primarily in degree (low, moderate, high), rather than in pattern. These findings indicate that, within this sample, autonomy, competence, and relatedness tended to covary, such that the satisfaction of one need was associated with satisfaction of the others. This pattern aligns with variable-centered SDT research showing that the three needs are interrelated (Ryan and Deci, 2019, 2022), suggesting that individuals who report satisfaction in one need often report similar satisfaction in the others. Supplementary analyses provided further support for a three-factor structure in which autonomy, competence, and relatedness are correlated yet empirically distinct constructs, rather than dimensions of a single need satisfaction factor. Although the profiles primarily reflected quantitative differences in overall need satisfaction, the person-centered approach remained valuable by identifying subgroups of employees who differed in their joint, overall level of autonomy, competence, and relatedness satisfaction. It also allowed investigation of whether different forms of job crafting were associated with membership in these low, moderate and high overall need satisfaction subgroups, addressing a research question distinct from the average associations estimated in variable-centered analyses.

A notable finding was that the moderate need satisfaction profile represented the largest group, comprising more than half of the sample. These average levels of need satisfaction likely reflect contextual realities within South African SMEs, where resource limitations and financial constraints often impede the implementation of formal management and human resources practices (Sungwa, 2025). As most employees reported moderate need satisfaction, organizations may consider encouraging proactive job crafting behaviors that are associated with differences in employees’ need satisfaction experiences. In this context, job crafting emerged as a particularly relevant factor in differentiating membership across the identified need satisfaction profiles.

The primary R3STEP analysis indicated that job crafting behaviors were associated with profile membership, but the pattern differed across the three forms of crafting. Task crafting distinguished the high need satisfaction profile from both the low and moderate profiles, while cognitive crafting distinguished all three profiles. Relational crafting distinguished the high and moderate need satisfaction profiles from the low profile but did not differentiate the high and moderate profiles. The Firth sensitivity analyses generally supported these patterns, although the high versus low comparison produced highly imprecise estimates and should therefore be interpreted cautiously. Specifically, the Firth results supported the moderate versus low distinction across all three job crafting dimensions, while the high versus moderate distinction remained significant for task and cognitive crafting but not relational crafting. Overall, cognitive crafting showed the most consistent association with profile membership, distinguishing all three profiles in the primary analysis and remaining supported across the relevant Firth comparisons.

The differentiated pattern across crafting dimensions suggests that job crafting is not uniformly associated with need satisfaction profile membership. This pattern is consistent with broader job crafting theory, which suggests that crafting behaviors can differ in both their focus and motivational orientation (Constantini, 2024). Bruning and Campion’s (2018) role–resource approach–avoidance model distinguishes active, improvement-focused approach crafting from avoidance crafting directed toward reducing, evading, or eliminating undesirable aspects of work. Thus, job-crafting behaviors should not be assumed to have uniform implications for employees’ psychological experiences at work. Instead, task, cognitive, and relational crafting represent distinct ways in which employees may actively enhance their work experience: by altering work activities, changing work-related relationships, or reframing the meaning of work, respectively (Slemp and Vella-Brodrick, 2014; Wrzesniewski and Dutton, 2001).

Cognitive crafting showed the broadest pattern, suggesting that how employees interpret and reframe the meaning of their work (Wrzesniewski and Dutton, 2001) may be particularly relevant to differences in need satisfaction profile membership. By enabling employees to view their work as personally meaningful and connected to valued goals and relationships, cognitive crafting may support the satisfaction of autonomy, competence, and relatedness needs. For example, employees may experience greater autonomy when their work feels self-endorsed and aligned with personal values; greater competence when they recognize the value or impact of their contribution; and greater relatedness when they perceive their work as benefiting or connecting them to others. This interpretation is consistent with the proposition that cognitive changes can influence employees’ work-related attitudes and behavioral efforts (Lan et al., 2026). It is also consistent with research showing that cognitive crafting can enhance work meaningfulness and, through meaningfulness, thriving at work (Wang et al., 2024).

Task crafting showed a more selective pattern of association with need satisfaction profile membership, distinguishing the high need satisfaction profile from the low and moderate profiles, but not differentiating the low and moderate profiles. Task crafting entails proactive changes to the number, type, scope, or manner in which employees perform their work tasks (Slemp and Vella-Brodrick, 2014; Wrzesniewski and Dutton, 2001). Through task-level changes, employees may create a better fit between the practical demands of their work and their strengths, values, preferences, and psychological needs. For instance, modifying the execution or sequence of work tasks may increase discretion and volition, thereby supporting autonomy. Expanding or modifying tasks to use existing capabilities or develop new ones may strengthen competence. Task crafting may also indirectly support relatedness where altered tasks allow employees to make a visible, meaningful, or valued contribution to colleagues.

Relational crafting showed a distinct pattern, differentiating the low need satisfaction profile from the moderate and high profiles but not differentiating the moderate and high profiles. This pattern suggests that relational crafting may be particularly relevant to differentiating employees experiencing generally low levels of need satisfaction from those experiencing at least moderate levels across the three psychological needs. Through relational crafting, employees proactively initiate or strengthen work-related interactions to improve collaboration, social support, or access to feedback (Slemp and Vella-Brodrick, 2014; Wrzesniewski and Dutton, 2001). Such behaviors may provide access to social and developmental resources that support employees’ broader need satisfaction experiences (Kerksieck et al., 2019; Noesgaard and Jørgensen, 2024).

The present pattern is broadly consistent with job crafting theory, which proposes that task, cognitive, and relational crafting represent distinct routes through which employees may alter the design, social environment, and meaning of their work (Wrzesniewski and Dutton, 2001). However, the findings extend research that has often examined job crafting in relation to single outcomes, such as work engagement, job satisfaction, meaningful work, or organizational commitment, by showing that the three dimensions were associated differently with employees’ profiles of autonomy, competence, and relatedness satisfaction. At the same time, the findings should not be interpreted causally. Given the cross-sectional design, need satisfaction may influence employees’ propensity to craft their work, job crafting may contribute to need satisfaction, or both processes may operate reciprocally over time. Longitudinal and experimental research is therefore needed to clarify the temporal and causal processes underlying these profile associations.

It was also hypothesized that employees whose basic psychological needs are better satisfied would show higher intrinsic motivation than those whose needs are less satisfied. Consistent with SDT, the findings showed a graded pattern in intrinsic motivation across the three profiles: employees in the high need satisfaction profile reported the highest levels of intrinsic motivation, followed by those in the moderate and low need satisfaction profiles, with all three profiles differing significantly from one another. Also consistent with SDT is that the satisfaction of the three basic psychological needs is associated with greater intrinsic motivation, as fulfilled needs make employees more volitional and more likely to internalize and enjoy tasks (Coxen et al., 2021; Van den Broeck et al., 2016, 2021). When employees feel more autonomous, capable, and connected, their work becomes inherently rewarding, which is associated with higher levels of intrinsic motivation. Thus, individuals in the high need satisfaction profile are more likely to experience work tasks as enjoyable and self-endorsed. In contrast, those in progressively lower need satisfaction profiles may lack the psychological conditions required for intrinsic motivation to emerge to the same extent. In support of this finding, a recent study found that employees in profiles characterized by high satisfaction of all three basic psychological needs reported higher levels of work engagement and meaning in their work (Heinimäki et al., 2026). Because engagement and meaningful work are positively associated with intrinsic motivation (Autin et al., 2022; Coxen et al., 2023), these findings provide indirect support for the expectation that employees with higher need satisfaction will also exhibit higher levels of intrinsic motivation.

The study has several theoretical implications. First, it supports a multidimensional view of job crafting by showing that task, cognitive, and relational crafting should not be assumed to have equivalent associations with employees’ psychological experiences at work. Second, the study extends SDT and job crafting research by applying a person-centered approach to need satisfaction. Rather than treating autonomy, competence, and relatedness as separate variable-level outcomes, the present study identified employee subgroups that differed in their overall level of need satisfaction and showed that job crafting dimensions were differentially associated with membership in these subgroups. Third, the results provide preliminary support for the proposition that job crafting may be need relevant through multiple routes: task crafting through modifications to work activities and opportunities for discretion and skill use; cognitive crafting through changes in work meaning and personal interpretation; and relational crafting through changes in access to social connection, feedback, and collaborative resources.

## 6 Practical implications

Based on the study’s findings, several tentative practical implications for SMEs can be identified. First, employees differed primarily in their overall level of basic psychological need satisfaction, rather than forming qualitatively different combinations of need satisfaction. This suggests that SME interventions may benefit from creating work environments that support autonomy, competence, and relatedness simultaneously, rather than focusing on a single need. This is consistent with SDT, which proposes that the three needs are interrelated and covary (Van den Broeck et al., 2016). However, longitudinal and experimental research is needed to determine whether changes in one need are accompanied by changes in the broader need satisfaction profile.

Second, most employees belonged to the moderate need satisfaction profile, suggesting potential scope for strengthening need satisfaction through supportive work environments. One possible avenue could be to examine need-supportive leadership behaviors, particularly in SMEs where managers typically maintain close and direct contact with employees. Recent studies have also highlighted the importance of colleagues in supporting employees’ basic psychological needs (Coxen et al., 2026), suggesting that interventions to enhance colleague support may warrant further investigation.

Third, the differentiated associations across task, cognitive, and relational crafting suggest that these forms of crafting may have different implications for employees’ need satisfaction experiences. Cognitive crafting showed the most consistent association with profile membership, while task and relational crafting were also associated with differences between profiles. This suggests that interventions should not assume that all forms of job crafting are equally relevant. Organizations could support constructive crafting by providing appropriate autonomy, developmental feedback, mentoring, and opportunities for collaboration, rather than placing responsibility for need satisfaction solely on employees. Recent experimental evidence from Glaas et al. (2026) further suggests that cognitive crafting interventions may improve meaningful work, job satisfaction, and work engagement. However, whether such interventions improve basic psychological need satisfaction remains unclear. Future longitudinal and experimental research should therefore examine whether job crafting interventions influence autonomy, competence, and relatedness satisfaction and contribute to transitions between need satisfaction profiles.

Taken together, these findings suggest potentially useful directions for practice in SMEs, while also highlighting the need for stronger longitudinal and experimental evidence before firm intervention recommendations can be made. In particular, the observed associations indicate that all three job crafting dimensions may be worthwhile targets for future intervention work in resource-constrained SME environments, but the present study cannot show whether encouraging these behaviors would actually improve employees’ need satisfaction.

## 7 Limitations and recommendations for future research

The present study should be interpreted in light of several limitations, each of which suggests directions for future research. First, although the latent profiles demonstrated satisfactory classification accuracy, the overall sample size was modest and the smallest profile comprised only 23 employees. Consequently, estimates relating to this subgroup should be interpreted with caution, as the relatively small profile may have reduced the precision of the multinomial logistic regression estimates. Future research should seek to replicate the identified profile structure and associated predictors using larger samples to establish the stability and reproducibility of the findings. Furthermore, although participants represented a range of industries, some sectors were less well represented, which may limit the generalizability of the findings across different organizational contexts. The sample was also not truly representative of the South African workforce, with a disproportionately large proportion of participants identifying as one ethnic group. Future studies should therefore include larger and more demographically and occupationally diverse samples and examine whether the three-profile solution and the pattern of job crafting predictors are replicated in SMEs across South Africa and internationally.

Second, the cross-sectional design did not permit causal inference or analysis of profile transitions over time. Although the direction from job crafting to need satisfaction profile membership is theoretically grounded (Huyghebaert-Zouaghi et al., 2022; Slemp and Vella-Brodrick, 2014; Toyama et al., 2022), the relationship is likely reciprocal: employees who already experience higher need satisfaction may feel more resourced and motivated to craft their work (Olafsen et al., 2025). Future research should therefore employ longitudinal designs with multiple measurement waves to test cross-lagged relationships between crafting dimensions and need satisfaction profiles, and latent transition analysis.

Third, this study examined only basic psychological need satisfaction, whereas the SDT literature highlights the importance of considering need frustration and need unfulfillment as distinct constructs that are not simply the opposite of satisfaction (Huyghebaert-Zouaghi et al., 2021; Vansteenkiste et al., 2020). Incorporating these dimensions could yield a more nuanced understanding of employees’ psychological need experiences and may reveal additional or more complex profiles.

Fourth, the study relied on self-report measures for all constructs, which introduces the risk of common method variance. Future research would benefit from incorporating multi-source data, such as peer or supervisor ratings of job crafting behavior, or from adopting experience sampling designs that capture within-person fluctuations in crafting and need satisfaction over time. Such approaches would complement the between-person profile perspective adopted here and more fully capture the dynamic character of job crafting as conceptualized by Wrzesniewski and Dutton (2001).

Fifth, the recruitment strategy may have introduced selection bias, as participants recruited through social media, personal networks, SMEs, and business chambers may differ systematically from those not reached through these channels. The 15-month data-collection period (August 2024 to November 2025) may also have introduced temporal heterogeneity in participants’ responses. Future research should use broader sampling approaches and examine whether findings are consistent across data collection periods.

Finally, the LPA was conducted using factor scores derived from the CFA rather than estimating the measurement and profile models simultaneously. This two-step approach does not fully account for measurement uncertainty in the subsequent mixture model and may therefore affect profile enumeration and classification. Future research using larger samples should replicate these findings using fully integrated factor mixture modeling.

## Data Availability

The raw data supporting the conclusions of this article will be made available by the author upon request, without undue reservation.
